# Relationships between body composition, anthropometrics, and standard lipid panels in a normative population

**DOI:** 10.3389/fcvm.2023.1280179

**Published:** 2023-12-06

**Authors:** Marcus Weeks, Andrew D. Delgado, Jamie Wood, Bodi Zhang, Sarah Pesce, Laura Kunces, Loukia Lili, David Putrino

**Affiliations:** ^1^ Department of Rehabilitation and Human Performance, Icahn School of Medicine at Mount Sinai, New York, NY, United States; ^2^ Thorne HealthTech Inc., New York, NY, United States

**Keywords:** metabolic syndrome, lipids, body composition, lipid panels, biometrics

## Abstract

**Introduction:**

More than one third of adults in the United States (US) meet the clinical criteria for a diagnosis of metabolic syndrome, but often diagnosis is challenging due to healthcare access, costs and discomfort with the process and invasiveness associated with a standard medical examination. Less invasive and more accessible approaches to collecting biometric data may have utility in identifying individuals at risk of diagnoses, such as metabolic syndrome or dyslipidemia diagnoses. Body composition is one such source of biometric data that can be non-invasively acquired in a home or community setting that may provide insight into an individual's propensity for a metabolic syndrome diagnosis. Here we investigate possible associations between body composition, anthropometrics and lipid panels in a normative population.

**Methods:**

Healthy participants visited the Lab100 clinic location at a hospital setting in New York City and engaged in a wellness visit led by a nurse practitioner. Blood was analyzed at point-of-care using the Abbott Piccolo Xpress portable diagnostic analyzer (Abbott Laboratories, IL, USA) and produced direct measures of total cholesterol (TC), high density lipoprotein (HDL-C), low density lipoprotein (LDL-C), very-low density lipoprotein (VLDL-C), and triglycerides (TG). Body composition and anthropometric data were collected using two separate pieces of equipment during the same visit (Fit3D and InBody570). Regression analysis was performed to evaluate associations between all variables, after adjusting for age, sex, race, AUDIT-C total score (alcohol use), and current smoking status.

**Results:**

Data from 199 participants were included in the analysis. After adjusting for variables, percentage body fat (%BF) and visceral fat levels were significantly associated with every laboratory lipid value, while waist-to-hip ratio also showed some significant associations. The strongest associations were detected between %BF and VLDL-C cholesterol levels (*t* = 4.53, *p* = 0.0001) and Triglyceride levels (*t* = 4.51, *p* = 0.0001).

**Discussion:**

This initial, exploratory analysis shows early feasibility in using body composition and anthropometric data, that can easily be acquired in community settings, to identify people with dyslipidemia in a normative population.

## Introduction

Independent of geographic, demographic and socioeconomic characteristics, approximately 1 in 3 adults in the United States (US) will avoid medical appointments that they deem to be necessary due to low health efficacy, difficulty accessing care, the cost of care, and the general dislike or discomfort of typical medical examinations (1). In addition, result turnaround in the primary care setting can be slow and an estimated 3.5%–20% of the US population (2) are needle phobic, which may lead to avoidance of medical evaluations or treatment. As health technology continues to progress, there is an increasing interest in using non-invasive biometrics that can be acquired in non-traditional medical settings to predict health status and overall health risk. These emerging technologies may be able to address a critical public health need to develop fast, accessible, and less invasive ways to capture actionable health data for modern healthcare consumers. Use of technologies for more accessible health testing has the potential to be applied to many conditions that currently impose important health concerns nationwide. For example, in 2012 more than a third of adults in the US met the clinical criteria for a metabolic syndrome diagnosis (3), setting the scene for a major public health issue. A significant challenge associated with addressing metabolic syndrome on a population health level is the fact that many patients are often unaware of their diagnosis and risk. For instance, as an important dimension of the metabolic syndrome diagnosis, regular monitoring of dyslipidemia is recommended (4), but since blood tests are the only way to gather information about dyslipidemia, many people do not engage in the necessary testing. A potential solution to address these barriers is to explore less invasive and more accessible ways to capture important biometric data, such as regional body composition, which has shown strong associations with metabolic syndrome (5).

Traditionally, clinical anthropometry consists of performing systematic measurements of the human body to detail both its size and shape (6). Similarly, body composition analysis uses technologies such as dual energy x-ray absorptiometry (DEXA) to return metrics such as relative percentages of bone mass, muscle mass, visceral fat and other types of soft tissue that make up total body mass (7). Dyslipidemia has been studied previously in relation to regional body composition namely, android (central adiposity) and gynoid (adiposity at the hips). One such study using DEXA found that a higher android to gynoid fat ratio was predictive of CVD and dyslipidemia (8). While DEXA has proven to be a valid and reliable gold-standard for gathering both body composition and anthropometric data, the required technology is expensive, occupies a large physical footprint, and requires significant training and expertise to operate regularly. Two alternatives to the DEXA apparatus are 3D body scanning and Bioelectrical Impedance Analysis (BIA) technologies. Both of these modalities are of comparable accuracy (9, 10), less invasive, less expensive, require less training and credentials to operate while providing instantaneous results. However, the utility of such low-cost and non-invasive technologies for predicting conditions such as dyslipidemia has not been adequately explored.

Thus, while standard blood chemistries have traditionally been used as the primary indicators of metabolic disease, there are many access barriers for members of the general population which leads to delays in identification and diagnosis of life-threatening conditions. If more individuals could be rapidly and less-invasively screened for indicators of metabolic disease, there would be great potential to prevent serious morbidity and mortality in the general population. The goal of this exploratory work is to evaluate whether meaningful associations between body composition, anthropometrics and blood lipid levels exist in a normative population, with the hopes of advancing predictive capabilities for serious health conditions through inexpensive, scalable, and minimally invasive technologies.

## Methods

### Ethics statement

All study procedures and analyses were fully approved by the Mount Sinai Program for the Protection of Human Subjects (IRB#: 17-00952).

### Data collection

Participants visited the Lab100 clinic location at a hospital setting in New York City. Each study visit was conducted by a nurse practitioner. A standard venipuncture blood draw was performed by the provider, which included testing for a full lipid panel. Participants were instructed to fast for at least 10 h prior to the blood draw and to consume a minimum of 12 ounces of water before arrival. The blood was analyzed at point-of-care using the Abbott Piccolo Xpress portable diagnostic analyzer (Abbott Laboratories, IL, USA) and produced direct measures of total cholesterol (TC), high density lipoprotein (HDL-C), low density lipoprotein (LDL-C), very-low density lipoprotein (VLDL-C), and triglycerides (TG).

Body composition and anthropometric data were collected using two separate pieces of equipment during the same visit (Fit3D and InBody570). The anthropometric assessment was completed using the Fit3D ProScanner (Fit3D, CA, USA). Following instructions to empty their bladder, participants changed into minimal tight-fitted clothing without shoes, socks, or jewelry and completed a scan. The scanner provided values for body geometrics including lengths, circumferences, areas, and volumes of the limbs, trunk and head. The Fit3D ProScanner's utility to produce body volume and composition metrics has been previously validated against a gold standard of Air Displacement Plethysmography (ADP) (9). Using data from the Fit3D, a body shape index (ABSI), surface-based body shape index (SBSI) and waist-to-hip ratio (WtHR) were calculated for each participant. Calculations for these variables are as follows:

$$\begin{array}{ccc} \mathrm{SBSI}=\frac{\left(H^{7/4})(WC^{5/6}\right)}{\left(\mathrm{BSA})(\mathrm{VTC}\right)}\; & \mathrm{ABSI}=\frac{\mathrm{WC}}{\left(\mathrm{BM}I^{2/3})(H^{1/2}\right)} & \mathrm{WtHR}=\mathrm{WC}:\mathrm{HC} \end{array}$$

Where *H* = Height; WC = Waist Circumference; BSA = Body Surface Area; VTC = Vertical Trunk Circumference; BMI = Body Mass Index; and HC = Hip Circumference.

Next, participant body composition was analyzed per manufacturer instructions on the InBody570 (InBody USA, Cerritos, CA). The InBody 570 has been validated against DEXA, which is a gold standard of body composition measurement (10). InBody570 allows percentage quantification of body fat (%BF), visceral fat level (VFL), skeletal muscle mass (SMM), water and bone in participants.

### Statistical analysis

Descriptive statistics are reported for survey respondent demographics. Distributions were examined for normality using histograms and *Q*-*Q* plots. Results for all continuous variables are presented as mean (SD), categorical variables are as *n* (%). Linear regression was used to measure the associations of anthropometric variables on lipid panel biomarkers. Regression analysis was performed in two stages, first, to estimate zero-order associations, and second, to examine those same associations but also adjusting for age, sex, race, AUDIT-C total score (alcohol use), and current smoking status. The selection of variables used for the adjusted models was based on previous research which examined the relationship between body metric parameters and/or lipid panel values (11–16). All model assumptions were verified (17). When there were significant associations at the zero-order level, a comparison was made between the zero-order estimates and the adjusted estimates. A 10% change in the estimate indicated the presence of confounding as calculated via the following formula: $\delta =\;\frac{\mathrm{zero}\text{-}\mathrm{order}-\mathrm{adjusted}}{\mathrm{zero}\text{-}\mathrm{order}}$. Model performance for the zero-order models was assessed to indicate the quality of the anthropometric factors on predicting lipid panel biomarkers. Specifically, the *R*^2^ and Akaike information criterion (AIC) were used to assess model performance with the best model having the highest *R*^2^ and the lowest AIC. Due to multiplicity concerns a family-wise false discovery rate *p*-value correction was applied, raw *p*-values were not interpreted; “families” corresponding to each table's *p*-values as well as the group of *p*-values for the regression analysis. All hypothesis tests were two-tailed, and *p*_FDR _< 0.05 was considered statistically significant. In the regression analysis, only significant estimates were interpreted and had their change scores calculated. All analyses were conducted using *R* (18).

## Results

### Study group characteristics

A total of 347 participants completed the Lab100 assessment protocol, and following our screening process that eliminated those with an incomplete dataset or were actively taking medication for dyslipidemia, a total of 199 participants were included in this analysis. Study population demographics, body composition measurements, anthropometrics, and confounding variables used in the adjusted model are reported in Table 1.

**Table 1:** Study population demographics and variables of interest including results of standard lipid panels.

Total participants (*n*)	199
Age [mean (SD)]	39.76 (11.02)
Gender = Female (%) = Male (%)	101 (50.8) 98 (49.2)
Race = White (%)	165 (82.9)
ABSI score [mean (SD)]	0.08 (0.00)
SBSI score [mean (SD)]	0.11 (0.01)
Waist-to-hip ratio [mean (SD)]	0.87 (0.07)
Body fat percentage [mean (SD)]	24.03 (9.66)
Skeletal muscle mass [mean (SD)]	31.24 (7.46)
Visceral fat level [mean (SD)]	7.54 (4.76)
AUDIT-C score [mean (SD)]	5.27 (1.53)
Current smoking status = current smoker (%)	13 (6.5)
Cholesterol levels (mg/dl) [mean (SD)]	185.63 (31.08)
HDL-C levels (mg/dl) [mean (SD)]	63.97 (15.64)
LDL-C levels (mg/dl) [mean (SD)]	99.47 (28.85)
Triglyceride levels (mg/dl) [mean (SD)]	110.82 (62.49)
VLDL-C levels (mg/dl) [mean (SD)]	22.20 (12.48)

ABSI, a body shape index; SBSI, surface-based body shape index; AUDIT-C, Alcohol Use Disorders Identification Test; HDL-C, high density lipoprotein cholesterol; LDL-C, low density lipoprotein cholesterol; VLDL-C, very low density lipoprotein cholesterol.

Variables with no designated units were treated as unitless variables.

### Specific body composition metrics are good predictors of lipid levels

In the unadjusted, zero-order effects, only WtHR was correlated with every laboratory value measured, while ABSI was not found to be correlated with any. VFL, %BF, SMM, and SBSI were all associated with some metrics but not others. The most significant zero order effect was seen between SMM and HDL-C as for every 1-unit increase in SMM there is a 0.81-unit decrease in the average HDL-C levels (*t* = −5.91, *p* < 0.0001). Table 2 details all zero-order associations found.

**Table 2:** All significant zero-order associations between lipid levels and body composition metrics.

Variable of interest	Dependent variable	Estimate	Standard error	Test statistic	*P*-value
ABSI	Cholesterol levels	879.5	441.2	2	0.0714
ABSI	HDL-C levels	76	224.3	0.3	0.7604
ABSI	LDL-C levels	727.7	410.4	1.8	0.1014
ABSI	Triglyceride levels	343.5	895.8	0.4	0.7604
ABSI	VLDL-C levels	67.5	178.9	0.4	0.7604
SBSI	Cholesterol levels	117.2	318	0.4	0.7604
SBSI	HDL-C levels	629.7	153.7	4.1	0.0001
SBSI	LDL-C levels	−215.4	294.8	−0.7	0.5825
SBSI	Triglyceride levels	−1,501.8	630.5	−2.4	0.0341
SBSI	VLDL-C levels	−300.6	126	−2.4	0.0341
Body fat percentage	Cholesterol levels	0.6	0.2	2.8	0.0132
Body fat percentage	HDL-C levels	0	0.1	0.2	0.8467
Body fat percentage	LDL-C levels	0.4	0.2	1.9	0.0771
Body fat percentage	Triglyceride levels	1	0.5	2.2	0.0451
Body fat percentage	VLDL-C levels	0.2	0.1	2.2	0.0451
Skeletal muscle mass	Cholesterol levels	0.1	0.3	0.5	0.7469
Skeletal muscle mass	HDL-C levels	−0.8	0.1	−5.9	<0.0001
Skeletal muscle mass	LDL-C levels	0.6	0.3	2.1	0.0556
Skeletal muscle mass	Triglyceride levels	1.9	0.6	3.3	0.0039
Skeletal muscle mass	VLDL-C levels	0.4	0.1	3.3	0.0039
Visceral fat levels	Cholesterol levels	1.4	0.5	3.1	0.0056
Visceral fat levels	HDL-C levels	−0.4	0.2	−1.8	0.1014
Visceral fat levels	LDL-C levels	1.2	0.4	2.9	0.0120
Visceral fat levels	Triglyceride levels	3.2	0.9	3.5	0.0022
Visceral fat levels	VLDL-C levels	0.6	0.2	3.5	0.0022
Waist-to-hip ratio	Cholesterol levels	74.3	29.2	2.5	0.0252
Waist-to-hip ratio	HDL-C levels	−74.9	14	−5.4	<0.0001
Waist-to-hip ratio	LDL-C levels	93.6	26.8	3.5	0.0022
Waist-to-hip ratio	Triglyceride levels	278.4	56.3	4.9	<0.0001
Waist-to-hip ratio	VLDL-C levels	55.7	11.3	4.9	<0.0001

ABSI, a body shape index; HDL-C, high density lipoprotein cholesterol; LDL-C, low density lipoprotein cholesterol; VLDL-C, very low density lipoprotein cholesterol; SBSI, surface-based body shape index.

Following adjustments however, VFL and %BF were found to be associated with every laboratory value. WtHR only had some significant associations while SMM, SBSI, and ABSI had none. %BF showed the two most significant associations with VLDL-C (*t* = 4.53, *p* = 0.0001) and TG (*t* = 4.51, *p* = 0.0001). Of the 13 total adjusted associations that were found, 11 of them had >10% change in estimated value as compared to the zero-order effect and thus indicated confounding was present and the adjustments were warranted (Supplementary Table S1).

## Discussion

Recent trends in preventative healthcare have shown a change in patients' preference towards clinical assessments in non-traditional settings that can improve access to care, provide a comfortable experience, non-invasive methodology and high-accuracy data predictions with meaningful and actionable results for their health and wellbeing. With the next generation technology and the wealth of information, this is feasible now more than ever. For example, important biometrics that are easy to assess accurately with the latest instruments can be used to give insights into one's risk for metabolic syndrome and dyslipidemia, predict mortality and morbidity and suggest dietary or lifestyle changes to enhance one's quality of life.

The present study explored associations between readily attainable anthropometric and compositional body metrics with standard lipid panel values. Of note, three specific body composition metrics, VFL, %BF, and WtHR, served as relatively strong predictors of lipid numbers. Other studies have attempted to determine significant associations using these metrics. In a Taiwanese population, both TGs and blood pressure were shown to be increased with increased VFL while there was a decrease seen in HDL-C values with increased VFL. Higher VFL was also associated with the presence of metabolic syndrome (19). Additionally, body fat has been assessed in specific populations and shown to be predictive of H-type hypertension (i.e., essential hypertension with elevated homocysteine levels), in both men and postmenopausal women (20, 21). Despite previous exploration of WtHR as a metric that may be predictive of cardiovascular disease, to date, no studies or meta analyses have shown such strong associations between VFL, %BF and WtHR with lipid values (22). Given that technological advances have made it possible for these metrics to be captured in locations such as gyms, pharmacies and other non-medical facilities, they may have significant triage utility in identifying people who should be referred for more invasive blood testing.

Interestingly, ABSI did not predict well in either the unadjusted or the adjusted models. This may be due to the well-known limitations that exist with tools such as BMI and WC, which are both used to calculate the ABSI measurement. BMI and WC, although used as screening tools for disease progression and weight management, seem to infer questionable insights about metabolic health (23). For all the predictive value that BMI has as a screening tool, it may mistakenly characterize individuals who have a higher than average fat-free mass, such as athletes, as being overweight or obese. Conversely, people with higher relative volumes of fat mass can be seen as healthier (24). Moreover, the sensitivity and specificity of BMI has been shown to be poor as compared to BIA as a predictor of obesity in both men and women with DEXA serving as the standard (25).

The variables that were used in the adjusted model were determined based on a number of previous literature and other studies assessing associations involving body metric parameters and/or lipid panel values. Visceral fat levels have been shown to increase with age in both men and particularly postmenopausal women (11, 12). Men generally have higher TG numbers while women have higher HDL-C levels (11, 13). Trends in cardiovascular risk factors show that in recent decades, black people in the US routinely have lower TC numbers despite having higher overall cardiovascular risk profiles (14). Additionally, alcohol consumption and cigarette smoking have effects on various components of the lipid panel (15, 16). Evaluation of our models pre- and post-adjustment for these variables also showed significant effects, indicating that our findings align with previous work that identify these variables as confounders of blood lipid profiles.

The main limitations of this initial exploratory study were the relatively small and homogenous nature of the study cohort. The reduced sample size was primarily affected by the need to remove participants who were on lipid modulating medications. This excluding criterion was necessary to remove factors that may affect associations between blood lipid levels and body composition/anthropometric measurements. In addition, the study population was rather homogenous in terms of race and ethnicity as well as geographical location. As such, although the results and trends are informative and statistically significant, follow-up trials that show reproducibility in a larger, more diverse, and longitudinal sample would be more representative of the general population. The direct impact of using body composition parameters on the barriers to risk stratification was not completed in this study, and is warranted to evaluate both the positive and neagative implications for using these methods. Finally, a healthcare cost analysis was beyond the scope of the present work, however will potentially provide valuable information on the financial benefits of using body composition metrics in the diagnosis of metabolic health if performed in the future.

## Conclusion

This work concludes that %BF, VFL, and WtHR are associated with most values in a standard lipid panel. Thus, body composition parameters have the potential to serve as an added, non-invasive, inexpensive, and scalable screening tool for assessing metabolic syndrome or other metabolic and lipid related health concerns. With such measures, there is the potential to improve the timeliness of patient diagnoses, optimize personalized treatment plans, and increase compliance of patient medical evaluations. Although this finding is statistically significant, larger and more diverse sample sizes are recommended to be analyzed in order to determine whether this proof-of-concept model is generalizable.

## Data Availability

The original contributions presented in the study are included in the article/**Supplementary Material**, further inquiries can be directed to the corresponding author.
