# Supplementary material for: Relationships between body composition, anthropometrics, and standard lipid panels in a normative population
Source: Front Cardiovasc Med. 2023 Dec 6;10:1280179. doi: 10.3389/fcvm.2023.1280179 (PMC10731366; doi:10.3389/fcvm.2023.1280179)
Supplement: Supplementary file 1 [file Datasheet1.pdf]

**Supplemental Materials**

**Supplemental Table. 1 Comparison of Effects**

| Variable of Interest | Dependant Variable | Unadjusted Estimate | Adjusted Estimate | Percent Change |
|---|---|---|---|---|
| Waist-to-Hip Ratio | HDL Levels | -74.92 | -49.32 | 34.17 |
| Waist-to-Hip Ratio | Triglyceride Levels | 278.4 | 249.11 | 10.52 |
| Waist-to-Hip Ratio | VLDL Cholesterol Levels | 55.71 | 49.39 | 11.33 |
| Visceral Fat Levels | Triglyceride Levels | 3.18 | 3.75 | -17.96 |
| Visceral Fat Levels | VLDL Cholesterol Levels | 0.64 | 0.75 | -17.68 |
| Visceral Fat Levels | Cholesterol Levels | 1.42 | 1.34 | 5.19 |
| Visceral Fat Levels | LDL Levels | 1.21 | 1.22 | -1.38 |
| Body Fat Percentage | Cholesterol Levels | 0.63 | 0.78 | -24.41 |
| Body Fat Percentage | Triglyceride Levels | 1.01 | 2.13 | -109.79 |
| Body Fat Percentage | VLDL Cholesterol Levels | 0.2 | 0.43 | -110.07 |
| Body Fat Percentage | LDL Levels | 0.41 | 0.7 | -71.36 |
| Visceral Fat Levels | HDL Levels | -0.42 | -0.62 | -49.58 |
| Body Fat Percentage | HDL Levels | 0.02 | -0.34 | 1615.94 |

**Gives values for the associations before and after adjustment with the model and their respective percent changes. Insignificant percent changes are indicated in red.**
